# Nonparametric Sparsification of Complex Multiscale Networks

**DOI:** 10.1371/journal.pone.0016431

**Published:** 2011-02-08

**Authors:** Nicholas J. Foti, James M. Hughes, Daniel N. Rockmore

**Affiliations:** 1 Department of Computer Science, Dartmouth College, Hanover, New Hampshire, United States of America; 2 Department of Mathematics, Dartmouth College, Hanover, New Hampshire, United States of America; 3 The Santa Fe Institute, Santa Fe, New Mexico, United States of America; University of East Piedmont, Italy

## Abstract

Many real-world networks tend to be very dense. Particular examples of interest arise in the construction of networks that represent pairwise similarities between objects. In these cases, the networks under consideration are weighted, generally with positive weights between any two nodes. Visualization and analysis of such networks, especially when the number of nodes is large, can pose significant challenges which are often met by reducing the edge set. Any effective “sparsification” must retain and reflect the important structure in the network. A common method is to simply apply a hard threshold, keeping only those edges whose weight exceeds some predetermined value. A more principled approach is to extract the multiscale “backbone” of a network by retaining statistically significant edges through hypothesis testing on a specific null model, or by appropriately transforming the original weight matrix before applying some sort of threshold. Unfortunately, approaches such as these can fail to capture multiscale structure in which there can be small but locally statistically significant similarity between nodes. In this paper, we introduce a new method for backbone extraction that does not rely on any particular null model, but instead uses the empirical distribution of similarity weight to determine and then retain statistically significant edges. We show that our method adapts to the heterogeneity of local edge weight distributions in several paradigmatic real world networks, and in doing so retains their multiscale structure with relatively insignificant additional computational costs. We anticipate that this simple approach will be of great use in the analysis of massive, highly connected weighted networks.

## Introduction

Network analysis has become a significant epistemological tool in the study of dense, complex relationship structures [1]. Sometimes the network structure is intrinsic to the relationships (e.g., the WWW [2], airline networks [3], or real neuronal networks [4]). In other cases it is a useful framework for codifying relationships of interaction such as those that might exist between organisms [5], social groups [6], [7], or technological structures such as the Internet [8].

In many important examples, network structure is imposed as a topological framework for enabling the useful visualization and subsequent analysis of a dataset (often “massive”) whose data points come *a priori* not as a network, but rather with a notion of similarity (or dissimilarity) between them. In these cases a weighted network can be used to summarize the similarity structure in the data as well as to provide a framework enabling exploration. For instance, this sort of structural assumption is the foundation of the spectral clustering approach to data analysis [9]. Important examples are “correlation networks,” in which the underlying data may be time series (see e.g., [10]), DNA microarrays [11], or any sort of multivariate feature vector, and the edge weights reflect a natural notion of correlation between the nodal data. These networks are generally “fully connected,” in the sense that there is a nonzero edge weight between (almost) any two nodes. Although the entire weighted network contains all relevant information, the important structure that exists within it often is not easy to recognize because the network itself is simply too dense with edges of nonzero weight. This problem is only amplified when the number of data points is large. This situation can pose problems, both for analytic methods (e.g., some spectral techniques) that may only be appropriate for a sparse network, as well as for visualization and the accompanying implications for gaining intuition from and communicating information about large data sets.

For these reasons it is often useful to extract the “backbone” of a network or to “sparsify” it by judiciously removing edges with the goal of elucidating underlying structure. Commonly, this is achieved through the application of a hard threshold, keeping only edges whose weight exceeds a predetermined value. In cases where the scale of the similarity values is constant across the nodes, such an approach will preserve meaningful geometry, insofar as this geometry is reflected in the connections between nodes with high similarity value. However, in real-world networks, similarity weight is often highly unevenly distributed. Consider the example of the Internet, where similarity between nodes can be defined as the bandwidth of the (direct) connection between them. Individual computers rarely are directly connected to the large lines that form the backbone of the Internet, and their connections through an Internet service provider will have bandwidth several orders of magnitude smaller than the highest bandwidth lines. If a threshold were applied to this network, the resulting set of connections would not accurately reflect the true underlying multiscale nature of the Internet. Indeed, only the highest-bandwidth connections would be preserved and all nodes in smaller bandwidth communities would be left as isolated points.

An alternative approach is to consider the local distributions of similarity weight and preserve edges that are statistically significant in a *local*, as opposed to a *global*, sense. Under such a model, the important connectivity of each node is considered separately from all others. This seems to have been first addressed by Slater in the context of the study of a directed graph of migration flows [12]–[14]. Therein we find the development and use of the so-called “bistochastic filter,” that aims to derive a single scale of importance by rescaling the edge weights via an iterative proportional fitting procedure (whose outcome is a doubly stochastic matrix) and then to create a backbone via the incremental addition of edges according to some global stopping criterion (e.g., fixed number of edges, fixed edge weight threshold, or connectivity constraint). In more recent work, local analysis has motivated the development and application of the “disparity filter” [5], wherein the statistical significance of edges at a particular node is determined relative to a null model obtained by dividing up the unit interval by throwing uniformly at random a set of points (of size equal to one less than the number of nonzero weighted neighbors of the node) onto the unit interval 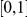. This provides a comparison set for the observed distribution of weights emanating from the node and a way to measure significance. As with any parametrized family of distributions, implicit is an *a priori* assumption about the shape of the distribution of similarity at any particular node. This approach is computationally very efficient and for some networks it can be quite useful.

While both of these methods can prove useful in particular instances, they each have their drawbacks. For example, the positing of a global constraint (in either the disparity or the bistochastic filter) ignores local heterogeneity. This can have the effect that the resulting network backbone may omit important multiscale structure.

With the drawbacks of previous approaches in mind, we make two important contributions to the analysis of complex networks. We show, for several paradigmatic weighted networks (including the US airline network [3] analyzed in [5]), that there is significant heterogeneity in similarity distributions, as mentioned above. Motivated by this, we present an approach to sparsification in which we *let the data speak for itself*: we use the empirical edge weight distribution at each node to determine statistically significant geometry. We show in the same set of examples that this approach, which makes no prior assumptions about the characteristics of the local distribution of similarity, adapts well to the heterogeneous nature of node-specific similarity distributions and at the same time preserves the important multiscale geometry of these networks. In three diverse “real-world” datasets we demonstrate that our method outperforms the disparity filter of [5] and the bistochastic filter [14] in its ability to recognize and account for the heterogeneity present in real-world networks. While these examples are specific, we see them as illustrative of the kinds of networks often encountered in real-world data. We anticipate that our approach to sparsification will be of great use in the analysis of massive, highly connected weighted networks.

### Backbone extraction and sparsification

Throughout, we assume that we are given a directed network with nonnegative edge weights 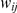 on the edge from node 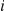 to node 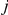. A traditional method for uncovering the underlying structure and visualizing such networks is to fix a threshold value 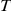 and retain all edges for which 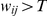. This method is straightforward to implement, but fails to retain important information when meaningful geometry is distributed across multiple scales of edge weights.

Another approach to capturing the multiscale structure of complex (similarity) networks is to find the *spanning tree* with maximal weight. A spanning tree of a network is a tree (a graph with no cycles) such that all nodes are connected and the edges that make up the tree exist in the original network. The weight of a spanning tree is the sum of the weights of all the edges that make up the spanning tree. Efficient algorithms exist to compute the *minimum* spanning tree of a network [15]. The maximal weight spanning tree can be easily computed via transformation of the non-zero edge weights 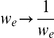, where 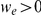 is the weight of edge 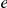, and then running a minimum spanning tree algorithm on the transformed network. The resulting maximum spanning tree keeps all nodes connected no matter the scale of the edges, but the imposed tree structure generally removes any community structure (clusters of nodes) that may be present.

The advantages and disadvantages of the previous methods offer some insight into the design of an effective network sparsification method for networks. There are obvious important requirements: we need an effective way to remove weak edges (as with thresholding), yet at the same time retain the most important edges at a local level (as in the maximal spanning tree). Furthermore, we want to allow important geometry such as cycles to persist so that communities remain intact.

The “disparity filter” of [5] was proposed as a method for backbone extraction that meets these criteria. Let
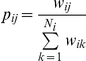
denote the *fractional edge weight* from node 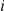 to node 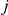. Here, 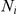 denotes the number of neighbors of node 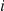 (for a weighted network, we say that 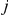 *is a neighbor of* 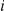 if 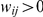). This edge distribution at node 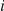 is compared to a null model in which 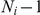 points are thrown down on the unit interval to create a random distribution of 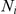 weights that sum to one. An edge is declared to be significant if the probability of observing an edge fraction 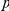 larger than 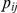 under the null model is less than some fixed value, i.e. 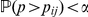 for a fixed 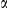. This approach is quite efficient and was applied by [5] to the US airline network [3] and the Florida Bay Food Web [16].

The “bistochastic filter” is another approach to sparsification, introduced in the study migration flows [12]–[14]. Through an iterative procedure, this method accomplishes a rescaling of the original edge weights matrix, creating a bistochastic matrix in which all columns and rows sum to one [17]. Once these transformed weights are obtained, edges are added to the network in order of non-increasing weight until a stopping criterion (such as a specific number of edges) is met. While this procedure does eliminate any multiscale structure in the weights themselves, it preserves certain important properties of the original matrix [14]. It is important to point out that the iterations do not always converge – see the discussion of the airline network example below and [14] – and in this case the algorithm is executed on a modified weight matrix. However, such a modification may in fact significantly alter the original network structure.

Both of these approaches can be useful for some kinds of networks. However, the underlying assumptions of each can in many instances result in a backbone that omits important structure – especially when the local weight distributions are highly heterogeneous. It is this concern that motivates the approach presented below.

## Materials and Methods

As above, given the original positive edge weights 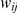, let 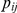 denote the corresponding fractional edge weight from node 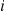 to node 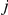. For such edge weight-normalized networks we will say that the *out-degree (resp. in-degree) of a node* is the number of positively weighted outgoing (incoming) edges. In the case of undirected networks, we simply consider the *degree* of a node to be the number of positively weighted edges incident to that node. For each node 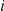 and all neighbors 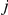, we consider the fraction of non-zero edges with weight less than or equal to 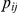


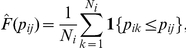
where 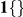 is the indicator function, 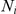 is the number of neighbors of node 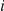 in the network, and the sum is over the neighbors of node 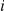. This calculation is effectively equivalent to the computation of the empirical cdf for the fractional edge weights at each node. For each edge, this gives the probability of choosing an edge at random of fractional weight less than or equal to 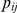. If 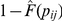 is less than a predetermined significance level 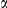, we say the edge is *locally significant* and include it in the backbone network. Thus, the edges that we select are ones that are statistically significant at the 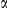 level and cannot be explained by random variation. If we desire a symmetric backbone network we may include the edge in the other direction as well. We call this method for backbone extraction *locally adaptive network sparsification*, or *LANS*. Note that we refer to LANS as a nonparametric method because it is a *distribution-free* method, in that no assumption is made as to the form for the underlying distribution of similarity weight at a node; instead, we rely only on the empirical distribution to judge statistical significance.

In Figure S4 we show pseudocode for generating backbone networks using LANS. Because we require the computation of the empirical cdf at each node, this algorithm runs in 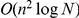 time, where 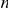 is the number of nodes in the network and 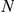 is the maximum node degree in the network. For networks with nonzero weight between any two nodes, 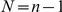, but in sparser real-world networks, 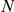 may be significantly smaller than 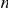. The asymptotic running time of the disparity filter method of [5] is 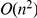, as is the basic step in the construction of the bistochastic filter (although, as an iterative algorithm it may not converge – see the example below of the airline network), and thus the running time of LANS is a factor of 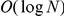 greater than either of the other approaches. Therefore, we pay a small asymptotic price in running time efficiency for the added flexibility of this algorithm. However, some of this cost can be mitigated by taking advantage of the fact that the algorithm is fully (and easily) parallelizable.

In order to understand the differences between LANS and the disparity and bistochastic filters, as well as the way in which real-world networks can display the heterogeneity we need to account for, we consider a toy network (described below) and three exemplary real-world weighted networks: a correlation network of equities composed of the S&P 500, FTSE 100, Nikkei 225, and Eurofirst 100 indices, the US airline network [3] (studied in [5]), and a similarity network of visual art. In the first example, the edge weight reflects the correlation between the “delta” time series of the daily closing price (that is, instead of closing price we use the one-period fractional return, 
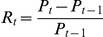
, where 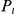 is the price of the stock at time 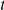) for equities over the period January 2, 2007 to December 31, 2009 [18]; in the second data set edge weights reflect passenger traffic between airports; in the last of these examples, images were summarized by feature vectors derived from a set of filters (learned via a sparse coding model) previously shown to be reflective of visual style [19] and with edge weights given by the symmetrized Kullback-Leibler divergence [20] between these feature vectors. Table 1 gives the number of nodes and nonzero edges for each of these data sets.

**Table 1:** Number of nodes and nonzero edges in each of the three real-world networks we studied.

	#Nodes	#Edges
Equities	874	381,501
Airlines	1256	13,239
Art	45	990

**Figure 1:**
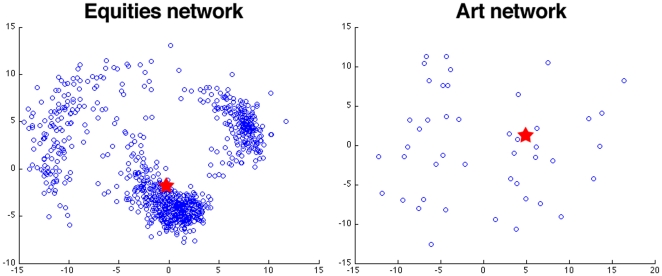
Demonstration of the heterogeneity of local fractional edge weight distributions in two weighted networks: equities from the S&P 500, Nikkei 225, FTSE 100, and Eurofirst 100 and the visual art network. The blue nodes represent the fractional edge weight distributions at each node in the network, the red star represents the corresponding null distribution produced by the technique of [5] (since the network is fully connected, there is a single comparison distribution). Distances are the output of a two-dimensional multidimensional scaling of the symmetrized Kullback-Leibler divergence between the distributions.

Figure 1 shows the heterogeneity in the local edge weight distributions for the equities network and the art style network. Specifically, for these example networks we computed each of the local fractional edge weight distributions. We also compute the distribution that would serve as the null model of [5] for each of these examples. Note that since both of these networks are fully connected there is a single distribution that serves as the null model for each of the nodes. We then computed a distance on this set of distributions by using the symmetrized Kullback-Leibler divergence between the distributions. Figure 1 presents the two-dimensional multidimensional scaling [21] scatter plots of the fractional edge weight distributions (blue circles) for our example networks and the disparity filter's distribution (red star), according to this pairwise distance. In each case the heterogeneous distribution of local edge weights is such that the parametric distribution is generally quite far from many of the local distributions. The Supporting Information provides a second measure of the heterogeneity of these weight distributions (relative to the disparity filter) via the Kolmogorov-Smirnov test (Table S1). In short, for each network, there is a significant amount of diversity in the empirical distributions. Moreover, they are judged as statistically different from the comparison parametrized distribution that produces the disparity filter. This particular test is not relevant for the bistochastic filter, since it does not consider local edge weight distributions when choosing which edges are added to the backbone network.

## Results

**Figure 2:**
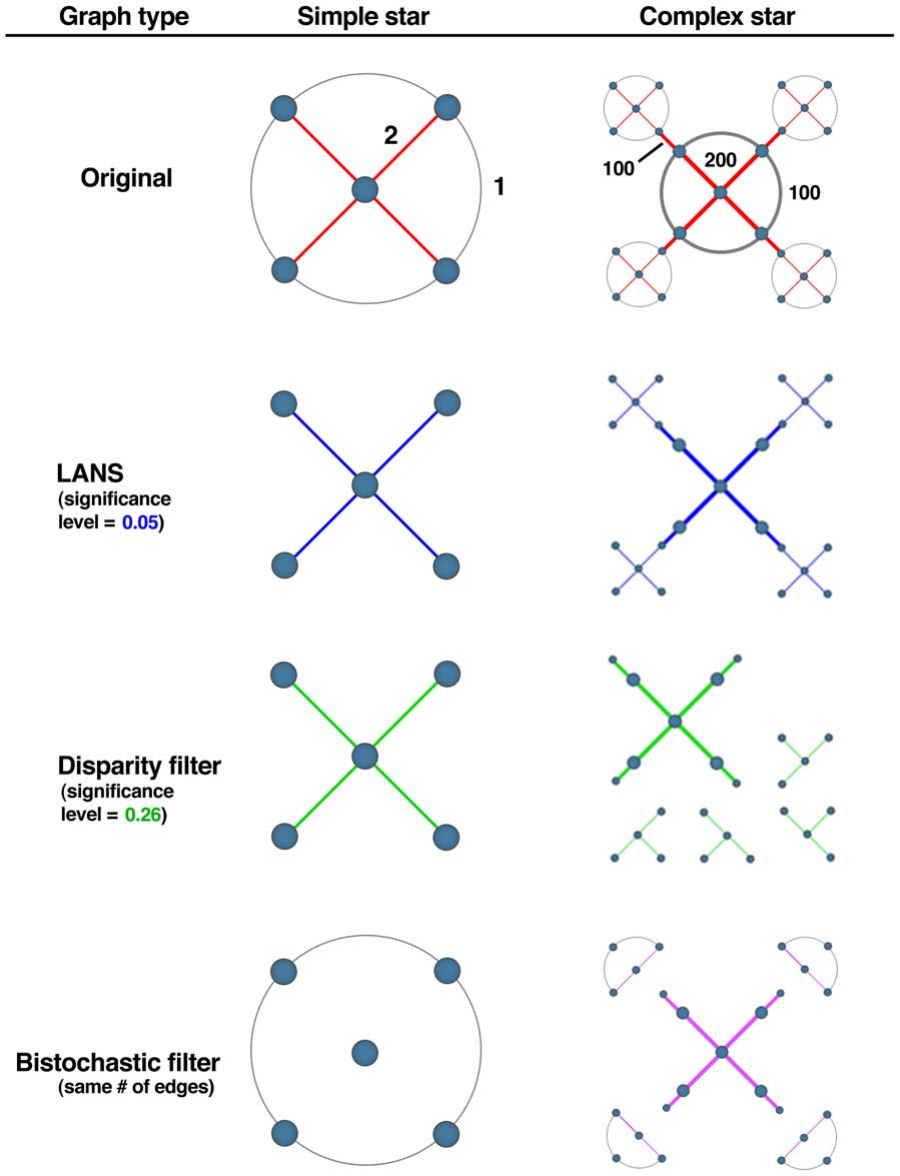
Two simple example networks with important geometry emphasized. In the “Simple star” network, the interior edges are emphasized by giving the edges in red weight 2 and the gray edges weight 1. LANS finds this significant geometry robustly across several values of 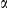 (0.05 chosen for demonstration). The disparity filter finds this geometry first at 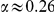. The bistochastic filter does not find the imposed geometry. In the “Complex star” network, the interior edges of each network are emphasized once again through differential weighting. However, the weights in the central network are two orders of magnitude larger than those in the peripheral networks. Red edges in the central network have weight 200, while gray exterior edges have weight 100. The red edges that formed the connections to the peripheral networks have weight 100. The peripheral networks have edges with weights 2 (red) and 1 (gray), as in the “Simple star” network. The disparity filter was applied to the “Complex star” network with the same value of 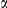 and the bistochastic filter method was stopped when the number of edges was equal to the number of edges in the network produced by LANS.

We start by comparing the application of LANS and the disparity and bistochastic filters in a motivating example: a simple weighted undirected network made from a combination of a star network and a ring, the “Simple star,” depicted in Figure 2. We assign weights to create a variation in which the higher weight edges are on the inner portion of the network. In particular, the red edges have weight 2 while the gray edges have weight 1. This adjustment emphasizes the “star” geometry in the network (i.e., a central node connected to a ring of outer nodes). In a backbone we would expect to recover the interior edges.

For this simple weighted network, LANS extracts the correct underlying geometry based on the differential weighting of edges in this network, and does so robustly across significance levels spanning several orders of magnitude. The disparity filter requires a very high significance level (i.e., large probability) to recover the expected geometry, and with stricter significance criteria the network is completely disconnected (i.e., no meaningful underlying geometry is discovered). A backbone network created using the bistochastic filter with the same number of edges as LANS included the edges that made up the “ring” geometry, but not the “star” geometry. This is a result of the iterative weight adjustment: the weak outer edges ultimately have a higher weight than the inner edges after the transformation, which clearly fails to capture the geometry that was emphasized. For this network, LANS provides a more flexible solution precisely because it does not assume a distribution on the fractional edge weights.

In order to test the capabilities of each of the methods to recover *multiscale* geometry in networks, we constructed a network out of several star/ring networks (the “Complex star” depicted in Figure 2), each with the star geometry emphasized. In this example, the central portion of the network is identical to the original “Simple star” network, but with edge weights 100 times those in the original network (yielding weights 200 and 100). The outer networks are copies of the simple network (with weights 2 and 1), and the connections between the central network and the peripheral networks have weight 100, as indicated in Figure 2. As before, the red edges highlight the important geometry in this network. This network is intended as a rudimentary analogue of the Internet. Using the same significance levels as with the Simple star network, LANS creates a backbone network that retains the correct multiscale structure of this network and preserves connectivity. Although the disparity filter does group nodes in an intuitive manner, it fails to preserve connectivity in this network. The bistochastic filter creates a backbone network (with the same number of edges as the two other examples) that retains the correct geometry in the central portion of the network, but incorrect geometry in the outer networks, as indicated by the edge colors in Figure 2. Once again, this occurs as a result of the weight transformation that does not emphasize the intended geometry.

Each of these approaches aims to extract backbones from networks whose degree distribution and edge weight distribution vary over several orders of magnitude. However, both the disparity filter and bistochastic filter ignore important local variation in edge weight. The disparity filter parameterizes local edge weight distributions according to node degree only, while the bistochastic filter ultimately ignores local information when choosing which edges to add to the network. In the case of the method of [5], there is an implicit assumption that the local edge weight distributions for nodes of the same degree in real-world networks are homogeneous. This implies that, for a given degree, there is a natural scale at which important geometry may exist that the method nevertheless fails to discover because it ignores the variability in the shape of local edge weight distributions – a variability that we will witness in the coming examples. This can be particularly problematic for fully connected networks, since nodes in these networks all have the same degree.

We will now explore the three real-world examples (the equities, airline, and art networks) in detail and see how LANS produces sparsifications that retain and expose the multiscale structure inherent in these networks.

### Equities network

**Figure 3:**
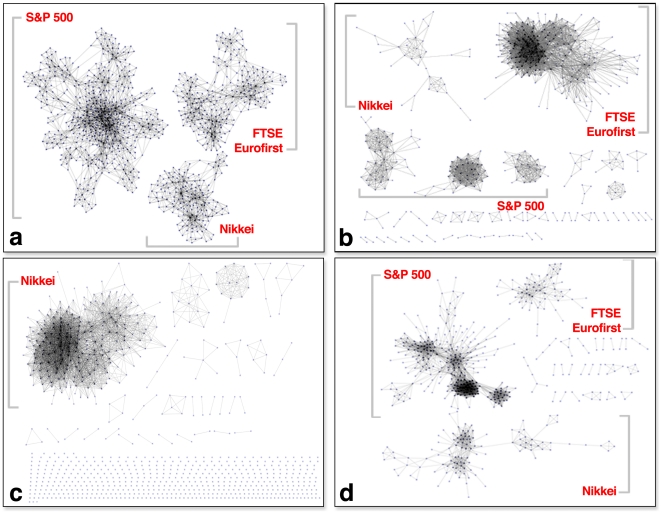
Comparison of four different sparsification methods for the equities market data. Box **a** shows the backbone created using LANS with significance level 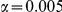. The backbone network shows three connected components, each of which correspond to the indices in which the stocks are present (with the two European indices fused). Box **b** shows the backbone created using the disparity filter [5], with 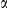 chosen to keep approximately the same number of edges. Box **c** shows the backbone network created using the bistochastic filter with the same number of edges as the network produced by LANS, and Box **d** shows a thresholded version of this network, with the threshold value once again chosen to retain approximately the same number of edges as in the network in Box **a**. The parametric backbone created using the disparity filter and the thresholded network retain some significant geometry, but fail to preserve even market-level connectivity. The backbone network produced by bistochastic filter contains a component that corresponds to the Nikkei and a few individual sectors, but most multiscale geometry has not been retained.

In Figure 3 we compare the results of applying LANS (Box **a**), the disparity filter (Box **b**), the bistochastic filter (Box **c**), and a hard threshold (Box **d**) to the similarity network derived from the equities data. Using 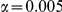 with the LANS method yielded a network with 3621 edges. The significance level for the parametric model (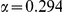) and the hard threshold (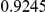) were chosen to retain roughly the same number of edges, and the bistochastic filtering process was stopped to include exactly this many edges. In (a) there are three connected components corresponding to the S&P 500, the Nikkei 225 and the combination of the FTSE 100 and Eurofirst 100 (which are both European indices). Within each component we see dense clusters of nodes which correspond to sectors (see below).

**Figure 4:**
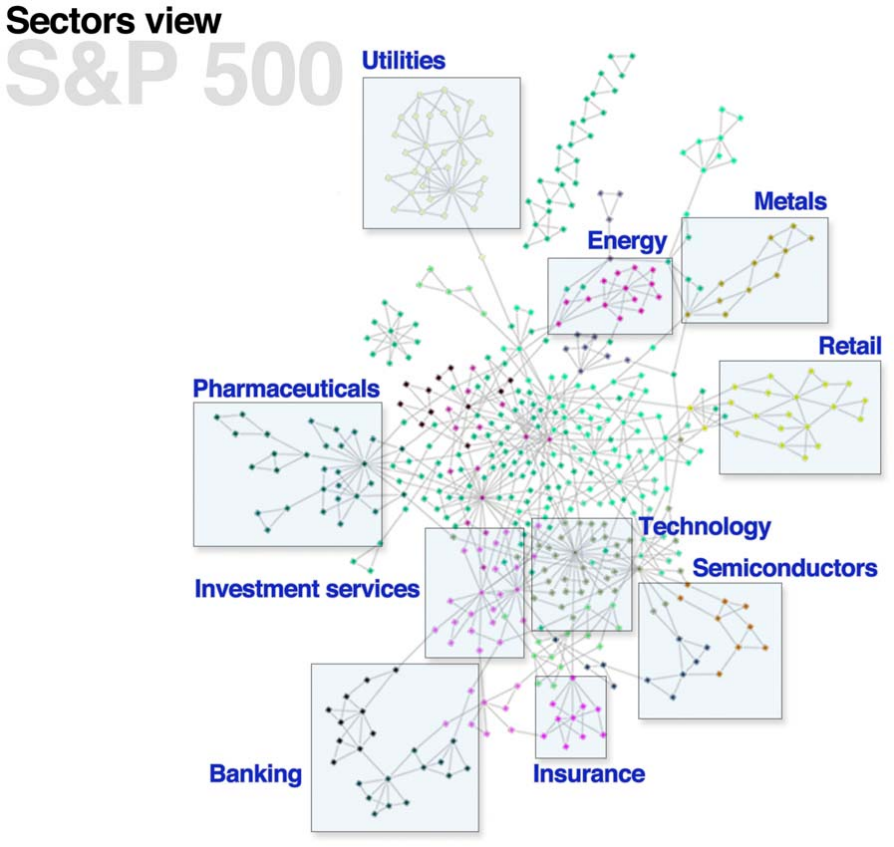
Clustering of the S&P 500 network (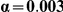) into 22 clusters using the spectral clustering algorithm [9]. Node colors indicate cluster membership. Some representative clusters have been labeled by industry and we see that the algorithm has done well at segmenting the network into groups of correlated stocks. In contrast, spectral clustering would have a hard time obtaining clusters of this quality from the complete network. We see that the nonparametric backbone is a useful preprocessing step for cluster analysis of networks. The number of clusters was chosen by using a Gaussian mixture model as part of spectral clustering and using the number of mixtures that maximized the likelihood of the data as the number of clusters.

In comparison, in Boxes **b**, **c**, and **d**, we see that while some market-level structure is retained in each sparsified network, many stocks are completely disconnected from any larger cluster of nodes. Furthermore, much of the large-scale structure is lost since the connected components of the networks in **b**, **c**, and **d** are both smaller and denser, and include only the connections between strongly correlated stocks while disregarding weaker, but significant, inter-cluster information. This reflects the fact that both the disparity filter and thresholding possess a natural scale that is inflexible to the actual distribution of fractional similarity weight on nodes, while the bistochastic filter simply does not consider local information when adding edges. In contrast, LANS, a nonparametric technique, uncovers the underlying geometry at multiple scales (markets all the way down to sectors and individual stocks), as opposed to retaining only the geometry corresponding to the largest correlations. To demonstrate the ability of LANS to capture local structure, we also created a backbone network on the S&P 500 equities only, using the LANS method with 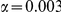, shown in Figure 4. The nodes of this network are colored according to the cluster assignment found via spectral clustering with 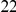 clusters (see Text S1 for more detail on the method). LANS clearly captures intuitive local structure (i.e., market sectors) within the larger equities network, and we have identified several clusters that correspond to commonly identified sectors.

**Figure 5:**
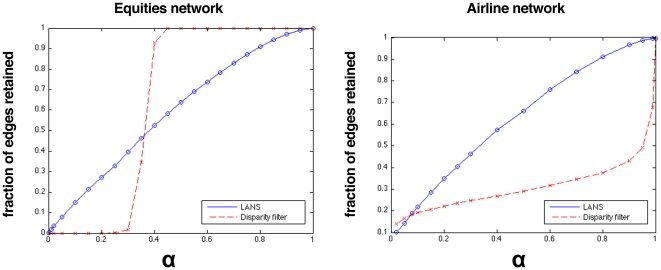
Fraction of edges retained in the equities network (left panel) and airline network (right panel) as a function of 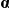. In the equities network the number of edges retained by LANS (circles) exhibits an almost linear trend, while that of the disparity filter (crosses) resembles a step function. With the disparity filter we witness a sharp increase in the complexity of the backbone network when a certain value of 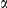 is reached, and very quickly the backbone becomes the entire network. Using LANS, as we increase 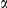, complexity is added gradually, allowing the extraction of structure at whatever scale is desired. The same linear relationship between 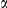 and the number of edges added by LANS exists in the airline network. The disparity filter adds edges in a non-uniform way: sub-linearly at first, then very quickly as 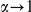.

A primary reason that an inflexibility to scale and local heterogeneity is so problematic is that it does not allow for smooth variation in the number of edges retained in the backbone network. This can be seen in the left panel of Figure 5, which shows the fraction of total edges in the equities network retained by LANS and the disparity filter as a function of 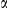. When using LANS, increasing 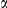 causes a smooth, linear increase in the number of edges in the backbone network, while the disparity filter provides a very small viable range in which the number of edges can be varied (below which there are no edges and above which the network is fully connected). Within this range, the number of edges increases at least exponentially. We also explored how edges were retained using the bistochastic filter by varying the edge weight threshold. In the equities network, edges were removed at an exponential rate, once the threshold was only slightly above zero (see Figure S5 for more detail).

The value of 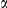 in LANS is a lower bound on the fraction of edges retained, and although local distributions vary, in practice we observed that the fraction of edges retained was well approximated by 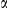 and grew linearly in 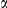 (as shown in the left panel of Figure 5). This smooth transition arises directly from the use of the empirical cdf of the distribution of fractional edge weight, since cutoff can vary not only by changing 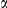, but also based on the characteristics of the fractional weight distribution itself. As we have shown in the example of fully connected networks, when using the disparity filter, the weight cutoff is determined for *every* node, once 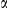 is chosen. This indicates that the LANS method possesses a level of flexibility that the disparity filter does not.

### Airline network

In practice, we find that LANS provides a more flexible solution than the disparity and bistochastic filters, even in real-world weighted networks that are *not* fully connected. As a second example, we considered the airline traffic network (measured in passengers traveling between airports), originally studied in [5], for U.S. domestic non-stop flights in the year 2006 [3]. This network represents the flow of airline passenger traffic around the U.S., and is not complete, since a direct connection does not necessarily exist between every pair of airports. We created a sparsified version of this network using LANS and the disparity and bistochastic filters (see Figure S3). The resulting backbone created using LANS had 1019 edges, and highlights the multiscale nature of this network: large numbers of passengers flow through important geographical/transportation centers (such as New York, Chicago, Atlanta, Dallas, etc.), and from these hubs to more distant locations. This is reflected in the tree-like structure of the network. The backbone network created using the disparity filter had 965 edges, roughly equal to the number of edges in the LANS version, but was unable to preserve much of the underlying connectivity in the airline network. Edges are added more quickly to clusters that are already connected, rather than between (more weakly connected) clusters. This leaves a great number of the nodes in this network completely disconnected from any larger component. The application of the bistochastic filter in this situation was somewhat problematic. In this case the iterative algorithm did not converge on the original matrix (which, as is mentioned in [14], can happen) and so in this case, the recommended approach is to augment the original weights by effectively inserting a small positive weight for transitions that were originally zero (this idea is revisited in the Discussion). This produced a backbone network that had tree-like structure, which would be expected, but without any multiscale features. Although some geographical localization was evident in this network, its meaning was not clear, unlike in the case of LANS (and to some extent the disparity filter) in which major cities are seen as connections between close-by airports and far-away destinations, as one would expect for airline traffic.

As with the equities network, we plot the fraction of edges retained in the backbone network as a function of 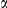 for LANS and the disparity filter (see Figure 5, right panel). Using LANS, the network exhibits a linear increase in the number of edges, whereas the disparity filter creates backbone networks that initially increase quickly in the number of edges, then sub-linearly, and finally exponentially as 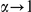. Using the bistochastic filter with an edge weight threshold, edges were removed exponentially quickly even when the edge weight threshold was only slightly above zero (see Figure S5 for more detail). Although for most values of 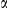, the disparity filter retained fewer edges, the actual backbone networks generated make it clear that this does not necessarily translate into a better description of the multiscale structure in this network. This is due primarily to the heterogeneity that exists in the local fractional edge weight distributions in this network (see Text S1 and Figure S2 for more detail). LANS adapts to this heterogeneity, retaining the most important geometry in such networks.

### Image network

The examples shown thus far have dealt with intuitive networks arising from a priori quantitative phenomena (stock price correlations, airline traffic); however, LANS is also useful for discovering important structure in feature-based representations of objects/phenomena. This type of data is common in machine learning and artificial intelligence applications, in which natural phenomena (such as images or speech audio) are transformed into a set of quantitative features in order to perform statistical calculations on them. As an example of such a dataset, we considered the similarity of the stylistic tendencies of a number of visual artists, and how these artists' works compare to images of the natural world.

In order to quantify “style” in this context, we measured the spatial frequency content in the images by examining the distribution of spatial bandwidths for a set of learned filters trained to optimally represent each artist's works (see e.g., [19], [22]). The bandwidths of these filters reflect the underlying distribution of spatial frequency content in each class of images (i.e., those by a particular artist). We computed the symmetrized (i.e., average) Kullback-Leibler divergence between the bandwidth distributions for all pairs of trained filters for these images (having trained five sets per image class).

**Figure 6:**
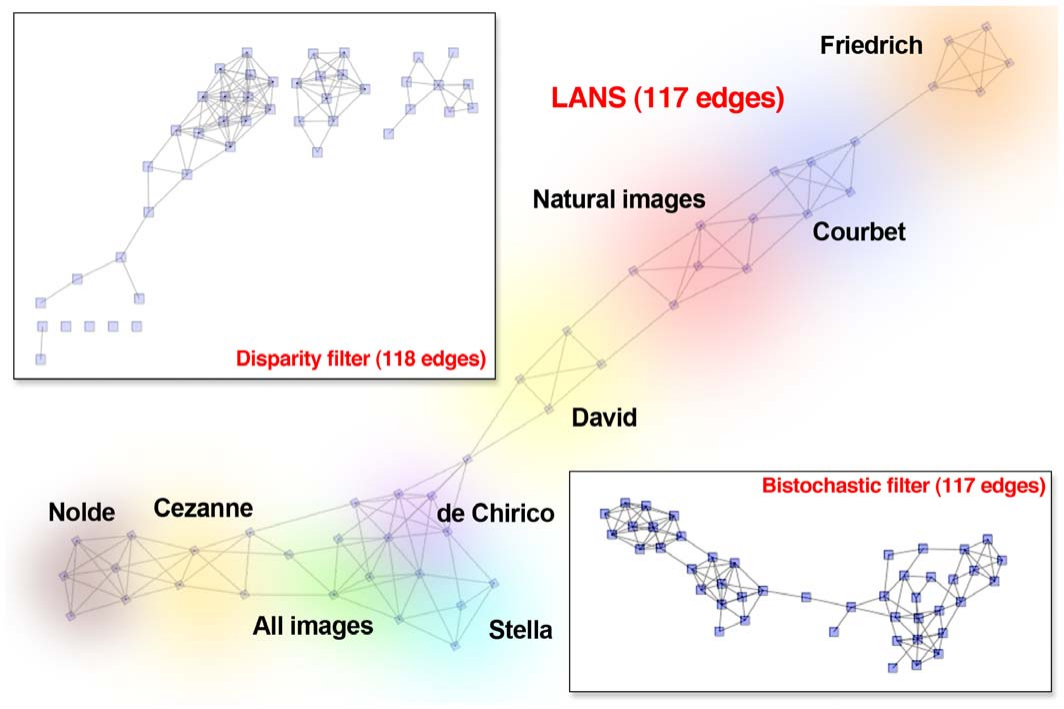
Art style backbone networks capturing the spatial frequency characteristics of several image classes created using LANS, the disparity filter (inset, top), and the bistochastic filter (inset, bottom). Each node represents one set of filters trained to optimally represent the corresponding image class, with five nodes per class, since five sets of filters were trained for each class. Image classes were composed of works by each of the artists indicated, as well as natural images and a grouping of all of the images together (the “All images” class). Similarity weight was derived from the symmetrized Kullback-Leibler divergence between the distributions of spatial frequency bandwidths for the learned filters. Clouds indicate the spatial arrangement of nodes in the network and that nodes in the same class were grouped together in this representation. The top inset shows the backbone network created using the disparity filter, with approximately the same number of edges as the LANS version. It is clear that, while some structure is retained, the relationships between image classes are largely ignored in the disparity filter version. Most of the important structure is retained in the backbone network created using the bistochastic filter, but local groups of nodes are denser with edges.

It was clear from visual examination of the similarity matrix derived from these values alone that the distribution of bandwidths was relatively consistent within a particular class (i.e., that filters trained on the same set of images had, as expected, highly similar distributions of spatial frequency bandwidth). However, the structure of the between-class similarity was not readily apparent from simple inspection of the raw values. Using LANS we created a sparsified network based on the derived similarity matrix (shown in Figure 6) in which each node represented a particular instance of a set of trained filters (and thus also its spatial frequency bandwidth distribution). The groupings of nodes were, as was evident from the similarity matrix, composed primarily of works by a particular artist. However, the geometry of this network indicated and reflected stylistic connections, after subjective examination of the division of clusters in the network, between artists based on how “painterly” they are. This quality refers to the extent to which brushstrokes themselves (as opposed to contours created by paint) are visible in an artist's work. Since the presence of visible brushstrokes would indeed affect the composition of spatial statistical structure in works of art, it is not surprising that this characteristic appears to affect the distribution of the bandwidths of the learned filters.

This example demonstrates the usefulness of LANS: whereas thresholding would have disconnected the individual clusters, masking the lower-level connections between them, LANS revealed an aspect of the relationship between these artists that was not otherwise apparent. Using the disparity filter, we created a backbone network with approximately the same number of edges and a backbone network using the bistochastic filter with exactly the same number of edges (both shown in Figure 6). Each of these methods retained the significant within-class node groupings that would be expected from the structure of the original data. However, the disparity filter nevertheless failed to highlight any multiscale structure that incorporated connections between classes. In the case of the bistochastic filter, multiscale interactions were captured, but once again at a price of denser connectivity between nearby nodes (which itself conveys little additional information).

## Discussion

We have presented a nonparametric, data-driven method, called locally adaptive network sparsification, for capturing the multiscale structure of weighted networks. We have shown that its flexibility adapts well to weighted networks in which local fractional edge weight distributions are highly heterogeneous, a quality that we have shown in three important and distinct real-world examples. Critically, LANS captures the multiscale structure present in these networks, preserving connectivity while including both local and global geometry. In each of the real-world examples we demonstrate that LANS outperforms the disparity filter method of [5] and the bistochastic filter of [14] in its ability to recognize and account for the heterogeneity present in real-world networks.

An interesting aspect that differentiates LANS from the disparity filter is that, in real-world networks, we observe an exponential increase in the number of edges in the network as 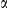 is increased when using the disparity filter (see Figure 5). In contrast, LANS adds edges roughly linearly as 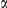 increases. An intuitive analogy that accounts for this phenomenon is that LANS extends the disparity filter in the way that Lebesgue integration extends Riemann integration [23]. That is, whereas the disparity filter implicitly uniformly partitions fractional edge weights (i.e., the domain of the distribution function), LANS uniformly partitions the *range* of the distribution function. This implies that, for LANS, there is a fixed lower bound for the difference in the distribution function between any two unique values of fractional edge weight, equal to 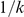, for a node with 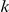 neighbors. Since the empirical cdf is monotonically increasing on [0,1] and by definition uses the entire interval, the value of the distribution function for each unique fractional edge weight falls into a bin of size at least 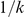. Thus, in order to incrementally add edges by unique weight, we need to consider steps of 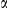 that are linear in the number of neighbors 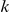 of a particular node. However, when using the distribution function specified in the method of Serrano et al., there are regions of fractional edge weight values in which the distribution function increases exponentially quickly. As a result, in order to incrementally add edges according to unique fractional edge weight, 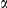 must be increased by an exponentially small value. This is a computationally impractical method for discovering the important underlying geometry in a network, and this problem is exacerbated by the fact that such tiny gradations of 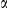 provide little additional benefit for large portions of the range of 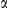.

A further discussion of computational aspects of each of the methods compared in this paper is warranted. The computational cost of both LANS and the disparity filter depends only on fixed quantities, namely the number of nodes in the network (both methods) and the maximal node degree (LANS only). The running time of LANS is 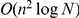, where 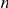 is the number of nodes in the network and 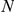 is the maximal node degree, while the running time of the disparity filter is 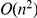. Since the bistochastic filter requires the use of an iterative algorithm to transform the edge weights, we can only discuss the cost of each iteration (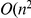)), but the number of iterations is possibly unbounded. In practice, we observed that when it converges, the iterative re-weighting algorithm converges relatively quickly; however, in some cases (e.g., the airline network), convergence does not occur and it is necessary to find an approximate solution by augmenting the original matrix [17]. This highlights another drawback of the bistochastic filter: there are a number of matrices for which the iterative weight adjustment algorithm will not converge, because they do not have an associated doubly stochastic matrix [17]. Methods such as adding infinitesimal weights to zero-valued entries in the weight matrix can be used to overcome certain difficulties, but at the cost of altering the original weight matrix. In cases where edge weights vary over several orders of magnitude and can be quite small, such an approach would cause a significant alteration in the underlying geometry of the network.

Almost all of our examples have great heterogeneity in the edge weights. In the case of a network with highly *homogeneous* edge weights, the disparity filter will provide an increasingly accurate model of fractional edge weight distributions (see Figure S1). It is not clear that this is the case for the bistochastic filter – note that in the “Simple star” example network, with its relatively homogeneous edge weights, the bistochastic filter did not articulate the expected geometry. It is important to recognize, however, that even in this case LANS will still correctly capture the uniform nature of these distributions, since it adapts to the shape of the distributions, regardless of their form.

In the future, we plan to extend the LANS approach to dynamic networks that evolve in time and to a probabilistic framework in which we learn posterior distributions over edges belonging to a backbone network. Furthermore, we will examine the connections between LANS, the disparity filter, and Markov Chains and will consider efficient implementations for large-scale data analysis (e.g., web-scale). We anticipate that LANS will be of great use in analysis and visualization of massive, highly connected weighted networks.

## Supporting Information

Text S1 Supplementary Text
(PDF)

Figure S1Comparison of the shape of the parametric model's cdf curves for varying values of 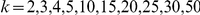 (node degree) going right to left. As 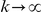, the cdf approaches a step function at 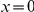.(TIFF)

Figure S2Comparison of cdfs for several randomly selected nodes in the equities (left) and airline (right) networks, along with the cdf for the parametric model. In each panel, the dashed, pink-colored line depicts the cdf for the parametric model. In the equities network, all nodes had degree 873, so the parametric cdf was parameterized according to this value. In the airline network, nodes had many different degrees, so we plot the cdf for all nodes with degree 51 and the corresponding parametric cdf. The 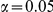 significance level is marked with the dashed line and indicates which edges would be retained at that significance level among the nodes. Any nodes with fractional weights to the right of the inverse of the cdf at the point it crosses the dashed line would be retained. It is clear that, at this significance level, no edges would be retained in the equities network using the parametric method. In the airline network, some of the empirical cdfs lie to the left of the model's cdf, and some to the right, demonstrating that, due to the heterogeneity of these distributions, the parametric model will add edges to the network in a non-uniform way.(TIFF)

Figure S3Box a shows the backbone network created using LANS with 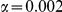. Although some nodes and small components are disconnected from the large primary connected component, the network itself is tree-like and exhibits the expected multiscale structure in the airline network (in particular, that major cities serve as connections for more outlying airports). Box b shows the backbone network created using the disparity filter with 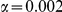, which had approximately the same number of edges as the network in Box a. Clearly, many more nodes are disconnected and the clusters that do exist are dense, indicating that this method tends to add edges to existing clusters, rather than form connections *between* them. Box c shows the backbone network created using the bistochastic filter. It does not retain any multiscale information (i.e., that large cities serve as hubs for airline travel).(TIFF)

Figure S4 Pseudocode for creating a backbone network using LANS.
(TIFF)

Figure S5 Fraction of edges retained as a function of edge weight threshold for backbone networks created using the bistochastic filter from the equities and airline networks. Note the exponential drop in the number of edges retained, indicating that the bistochastic transformation does not allow for a smooth addition of edges.
(TIFF)

Table S1Number of Kolmogorov-Smirnov tests that rejected the null hypothesis that the empirical distributions and the parametric distributions of [5] were the same, at the 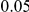 and 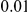 significance levels. The total number of tests for each network is given as well. We chose to compare the empirical distributions of fractional edge weight to the correct parametric cdf for all nodes in each network that had at least 40 unique nonzero edges. This number was chosen to ensure accurate results.(PDF)
