# Supplementary material for: Nonparametric Sparsification of Complex Multiscale Networks
Source: PLoS One. 2011 Feb 8;6(2):e16431. doi: 10.1371/journal.pone.0016431 (PMC3035633; doi:10.1371/journal.pone.0016431)
Supplement: Text S1 — Supplementary Text (PDF) [file pone.0016431.s001.pdf]

# Nonparametric sparsification of complex multiscale networks - Supporting Information Text S1

Nicholas J. Foti[1], James M. Hughes[1], Daniel N. Rockmore[1,2,3,*]
**1 Department of Computer Science, Dartmouth College, Hanover, NH, USA**
**2 Department of Mathematics, Dartmouth College, Hanover, NH, USA**
**3 The Santa Fe Institute, Santa Fe, NM**
**∗ E-mail: rockmore@cs.dartmouth.edu**

# Supporting Information Text S1

## Local heterogeneity

Supporting Information Figure S1 depicts a number of the distributions used in the disparity filter [1] for various node degrees. We see that the family of distributions is quite rich and able to capture many different shapes of the local fractional edge weight distributions. However, Supporting Information Figure S2 shows the local empirical cdfs for a random sample of nodes from the equities network (left panel), and the airline network (right panel). In the case of the equities network, all nodes have 873 neighbors (it is fully connected) and so a single distribution (thicker, pink-colored curve in figure) will be used by the disparity filter to determine significance. It is evident that the parametric distribution does not well-approximate the observed distributions. For the airline network we show the empirical cdfs of all nodes with degree 51, along with the distribution corresponding to the model of [1]. In this case the parametric distribution has similar shape to the empirical distributions; however, the shapes and scales of the empirical distributions vary, and it is evident that local information is being ignored.

As a quantitative measure to show the statistical significance of local heterogeneity we performed a Kolmogorov-Smirnov test [2] for all nodes in the equities, airline, and art networks with 40 or more neighboring nodes. The Kolmogorov-Smirnov test is a statistical test to determine equality of distributions between a sample (the empirical cdf) and a reference distribution (the parametric distribution [1] would have used for the node). Supporting Information Table S1 summarizes the results. We see that in the equities and art networks all tests rejected the null hypothesis at the 5% and 1% significance levels indicating the parametric distribution does not capture the true distributions of fractional edge weight. For the airline network 99.4% and 98.3% of the tests rejected the null-hypothesis at the 5% and 1% significance levels. Again we see that there is statistically significant heterogeneity in the distribution of fractional edge weight among nodes with the same degree. Therefore, the use of the empirical cdf in LANS is justified as it captures this extra information that previously has been ignored.

## Clustering equities with sparsified network

We evaluate network sparsification using LANS as a preprocessing step for a common task in network analysis, namely clustering nodes. The goal is to assign each node to a single cluster, such that nodes within the same cluster are "more similar" to each other than to nodes in other clusters. A common method for clustering the nodes of a network is with *spectral clustering* [3].

We perform clustering on the equities network sparsified using the LANS method with $\alpha = 0.003$. In Figure 4 in the main text of the paper, we have colored the nodes of the equities network according to the cluster assignment found with spectral clustering with 22 clusters. The number 22 was chosen as the number of clusters by maximizing the likelihood of a Gaussian mixture model on the same data. We see that most clusters do correspond to known economic sectors. This agrees with previous work on clustering equities using correlations [4].

### Airline network

Supporting Information Figure S3 depicts the sparsified airline network found with LANS (Box a), the disparity filter (Box b), and the bistochastic filter (Box c). We see that LANS has sparsified the network in such a way that more of the network is connected and there are far fewer very small components. Additionally, the resulting network is more tree-like. The backbone extracted using the disparity filter on the other hand has many more small components and the large component is much denser than that of LANS. This indicates that in this network LANS tends to add edges between connected components while the disparity filter tends to add edges within a connected component. The backbone network created using the bistochastic filter has a tree-like structure, but does not retain any multiscale information.

### LANS pseudocode

Supporting Information Figure S4 provides pseudocode for the creation of a backbone network using LANS, given a matrix $S$ of similarity values and significance level $\alpha$, return sparsified network in $A$.

### Additional tests using bistochastic filter

We performed two additional experiments using the bistochastic filter, intended to serve as a comparison with LANS. We measured the fraction of edges retained as a function of the edge weight threshold in the equities and airline networks and observed that most weights are quite small and thus are excluded exponentially quickly once the threshold rises even slightly above zero (see Supporting Information Figure S5). Thus, it is difficult to observe fine gradations between backbone networks generated using the bistochastic filter without using exponentially small increments in the weight threshold, which can be computationally very intensive.

We also compared our method to the bistochastic filter using a different stopping criterion for adding edges. Specifically, after obtaining a bistochastic matrix using the equities weight matrix, we kept all edges whose (transformed) weight exceeded the average of all the weights. This led to a very densely connected network with a single connected component and no obvious structure.

## Acknowledgments

## References

1. Serrano M, Boguna M, Vespignani A (2009) Extracting the multiscale backbone of complex weighted networks. Proceedings of the National Academy of Sciences 106: 6483-6488.

2. RO Duda PH, Stork D (2001) Pattern Classification. John Wiley & Sons, NY.

3. Hastie T, Tibshirani R, Friedman J (2009) The Elements of Statistical Learning. Springer, NY.

4. Leibon G, Pauls S, Rockmore DN, Savell R (2008) Topological structures in the equities market network. Proceedings of the National Academy of Sciences 105: 20589-20594.
