# Supplementary figures and images for: Nonparametric Sparsification of Complex Multiscale Networks
Source: PLoS One. 2011 Feb 8;6(2):e16431. doi: 10.1371/journal.pone.0016431 (PMC3035633; doi:10.1371/journal.pone.0016431)

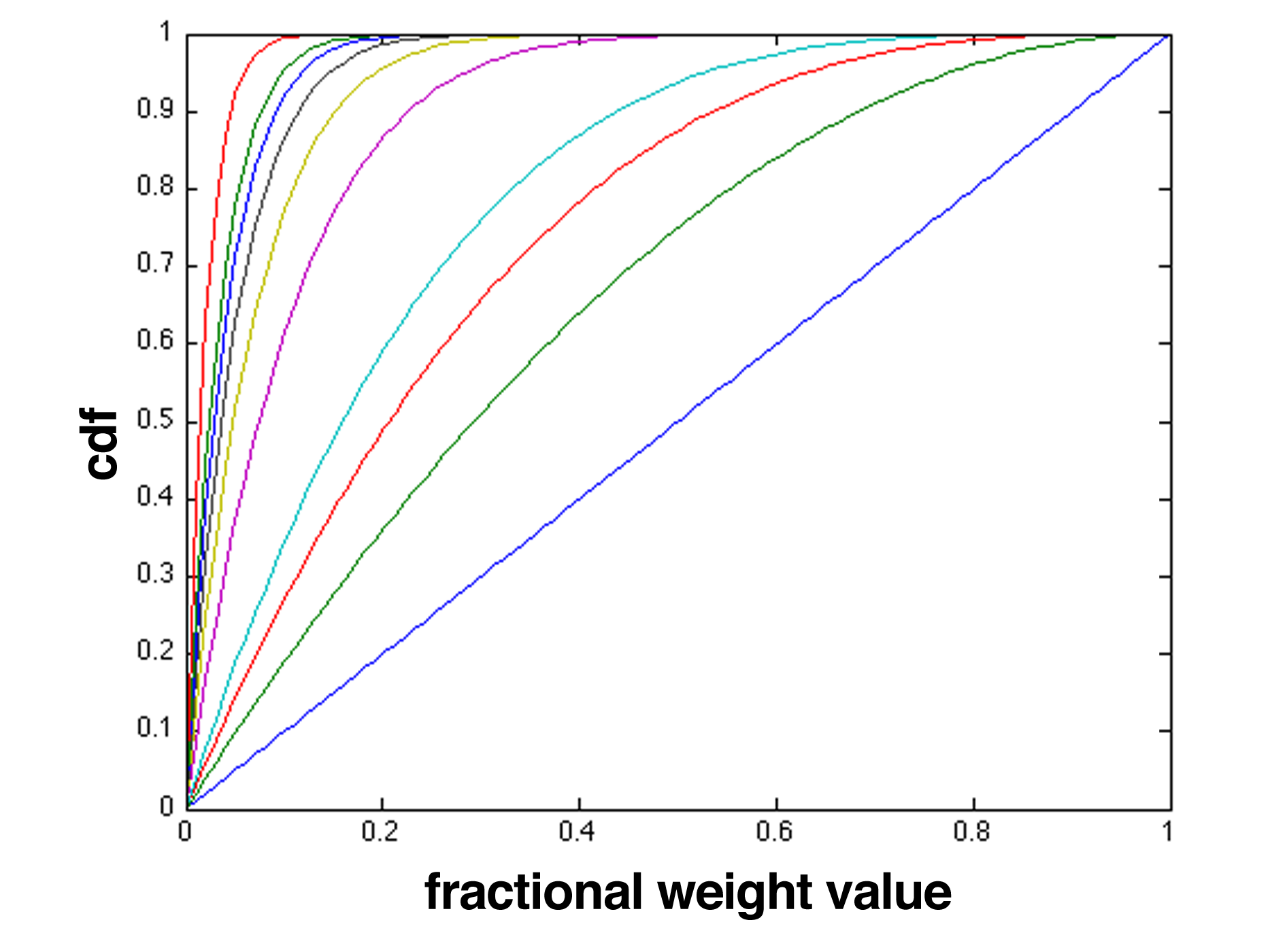

Supplement: Figure S1 — Comparison of the shape of the parametric model's cdf curves for varying values of (node degree) going right to left. As , the cdf approaches a step function at . (TIFF) [file pone.0016431.s002.tiff]

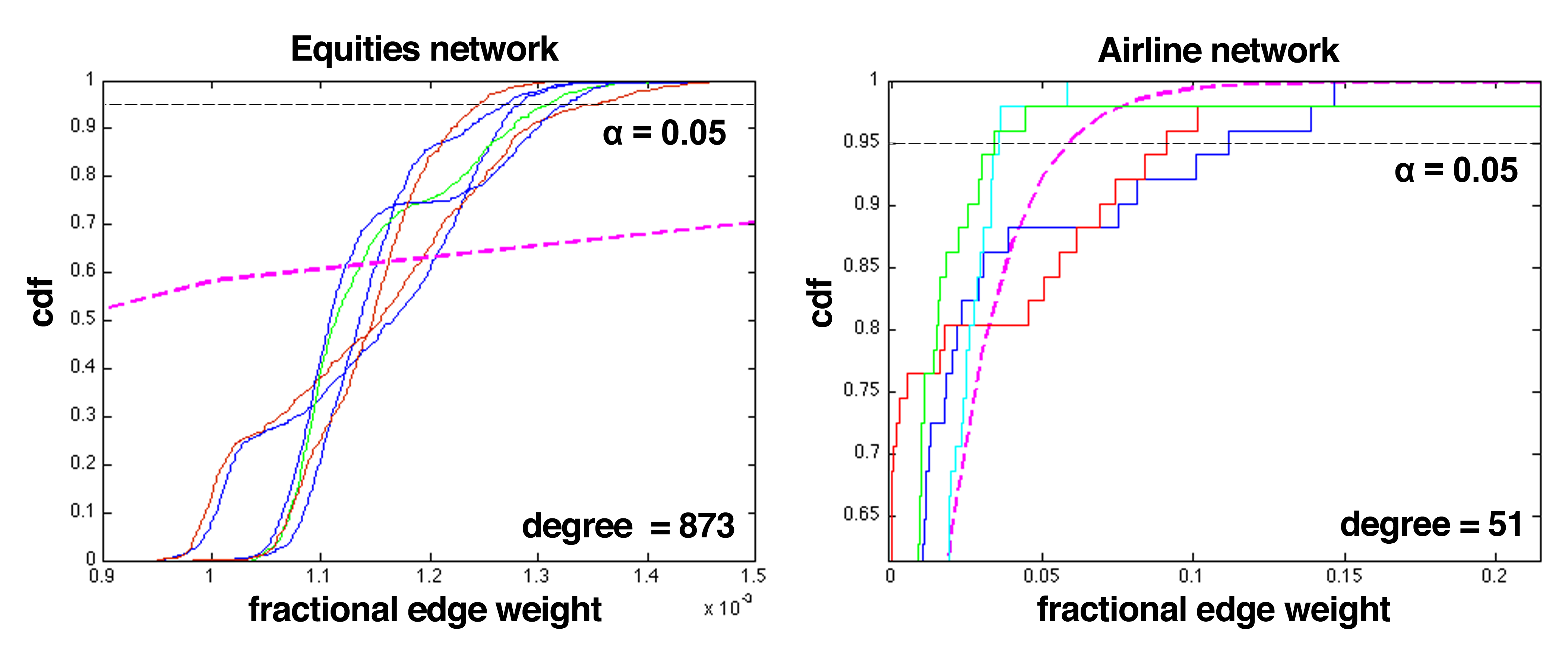

Supplement: Figure S2 — Comparison of cdfs for several randomly selected nodes in the equities (left) and airline (right) networks, along with the cdf for the parametric model. In each panel, the dashed, pink-colored line depicts the cdf for the parametric model. In the equities network, all nodes had degree 873, so the parametric cdf was parameterized according to this value. In the airline network, nodes had many different degrees, so we plot the cdf for all nodes with degree 51 and the corresponding parametric cdf. The significance level is marked with the dashed line and indicates which edges would be retained at that significance level among the nodes. Any nodes with fractional weights to the right of the inverse of the cdf at the point it crosses the dashed line would be retained. It is clear that, at this significance level, no edges would be retained in the equities network using the parametric method. In the airline network, some of the empirical cdfs lie to the left of the model's cdf, and some to the right, demonstrating that, due to the heterogeneity of these distributions, the parametric model will add edges to the network in a non-uniform way. (TIFF) [file pone.0016431.s003.tiff]

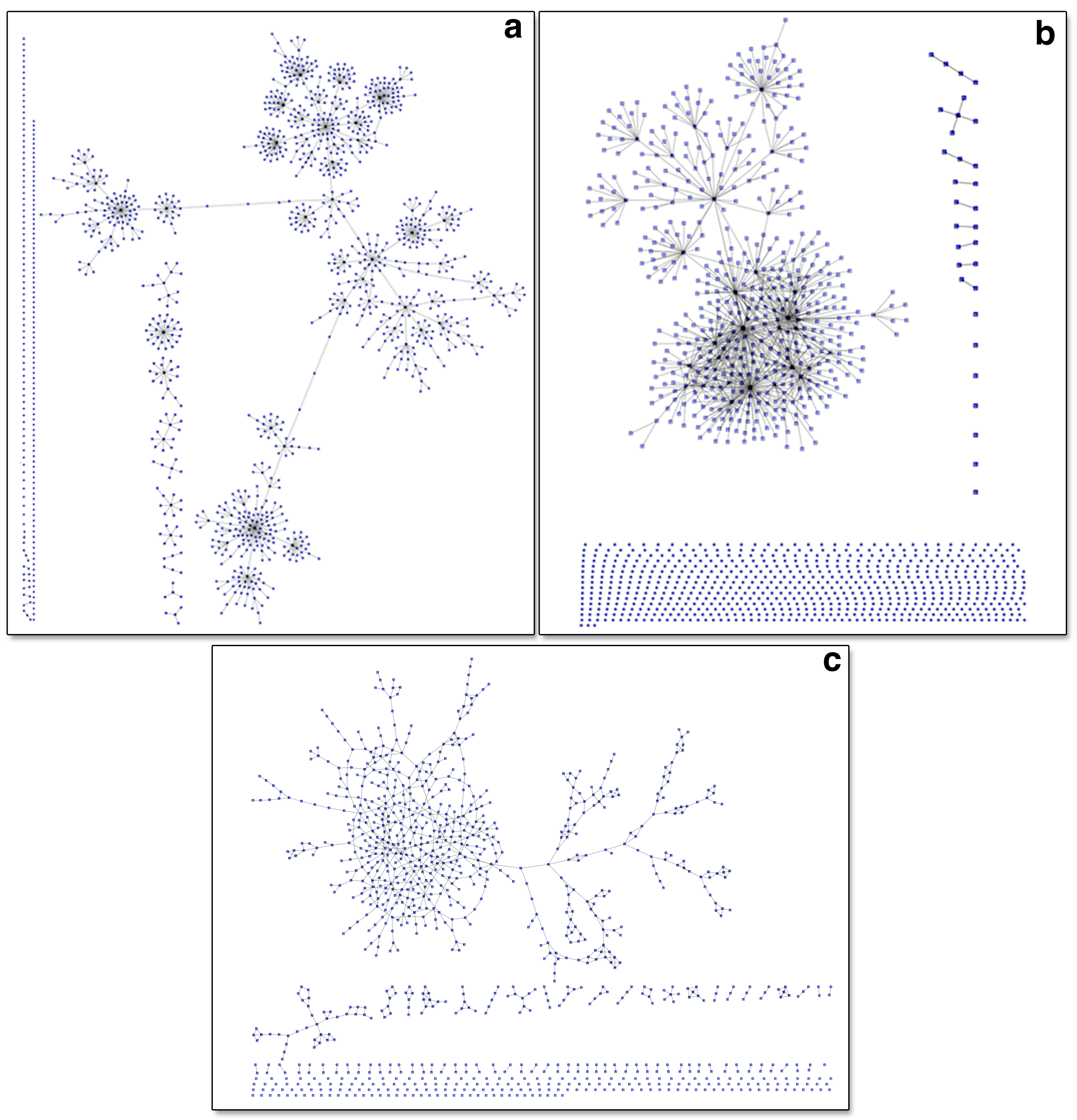

Supplement: Figure S3 — Box a shows the backbone network created using LANS with . Although some nodes and small components are disconnected from the large primary connected component, the network itself is tree-like and exhibits the expected multiscale structure in the airline network (in particular, that major cities serve as connections for more outlying airports). Box b shows the backbone network created using the disparity filter with , which had approximately the same number of edges as the network in Box a. Clearly, many more nodes are disconnected and the clusters that do exist are dense, indicating that this method tends to add edges to existing clusters, rather than form connections between them. Box c shows the backbone network created using the bistochastic filter. It does not retain any multiscale information (i.e., that large cities serve as hubs for airline travel). (TIFF) [file pone.0016431.s004.tiff]

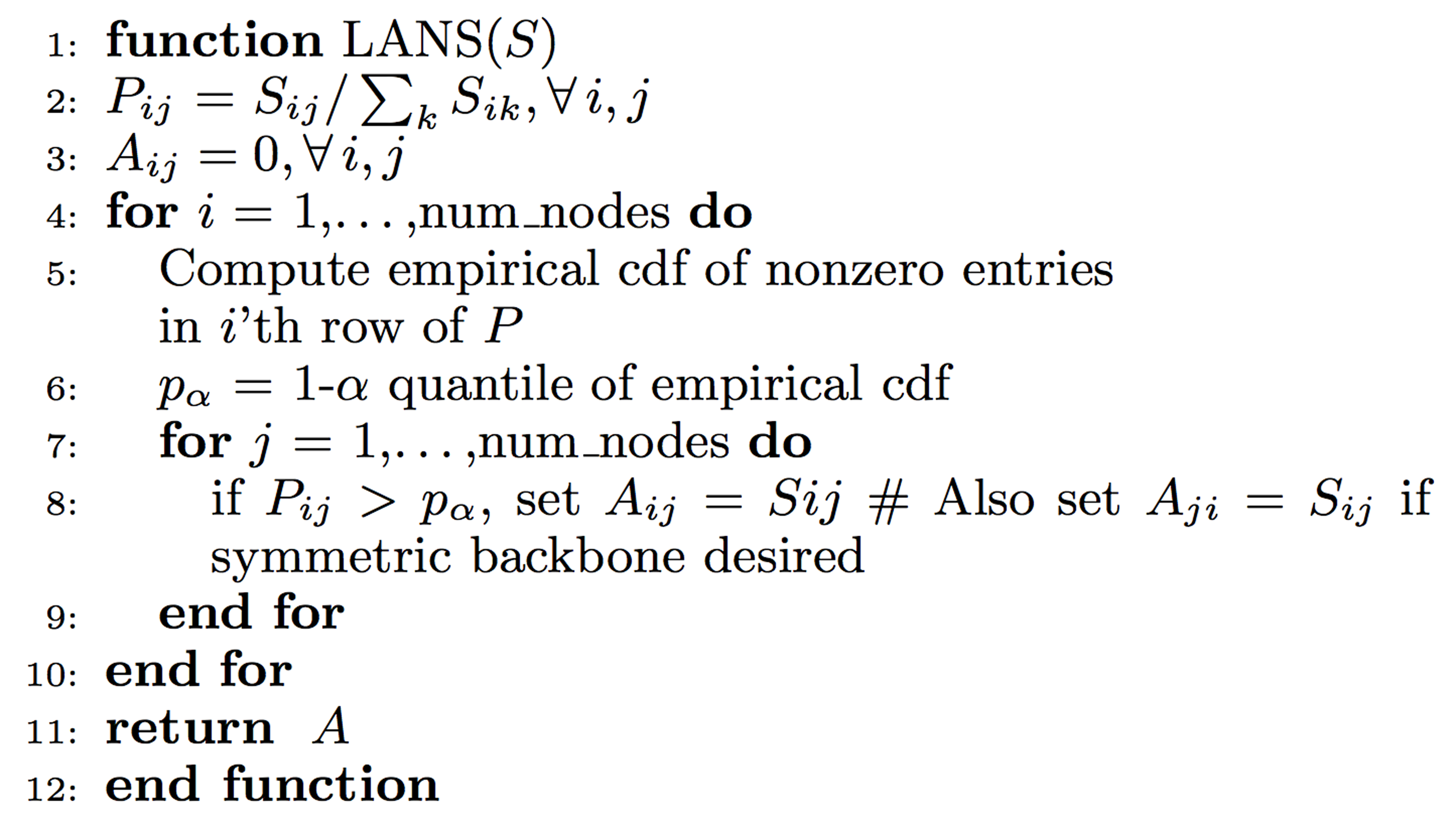

Supplement: Figure S4 — Pseudocode for creating a backbone network using LANS. (TIFF) [file pone.0016431.s005.tiff]

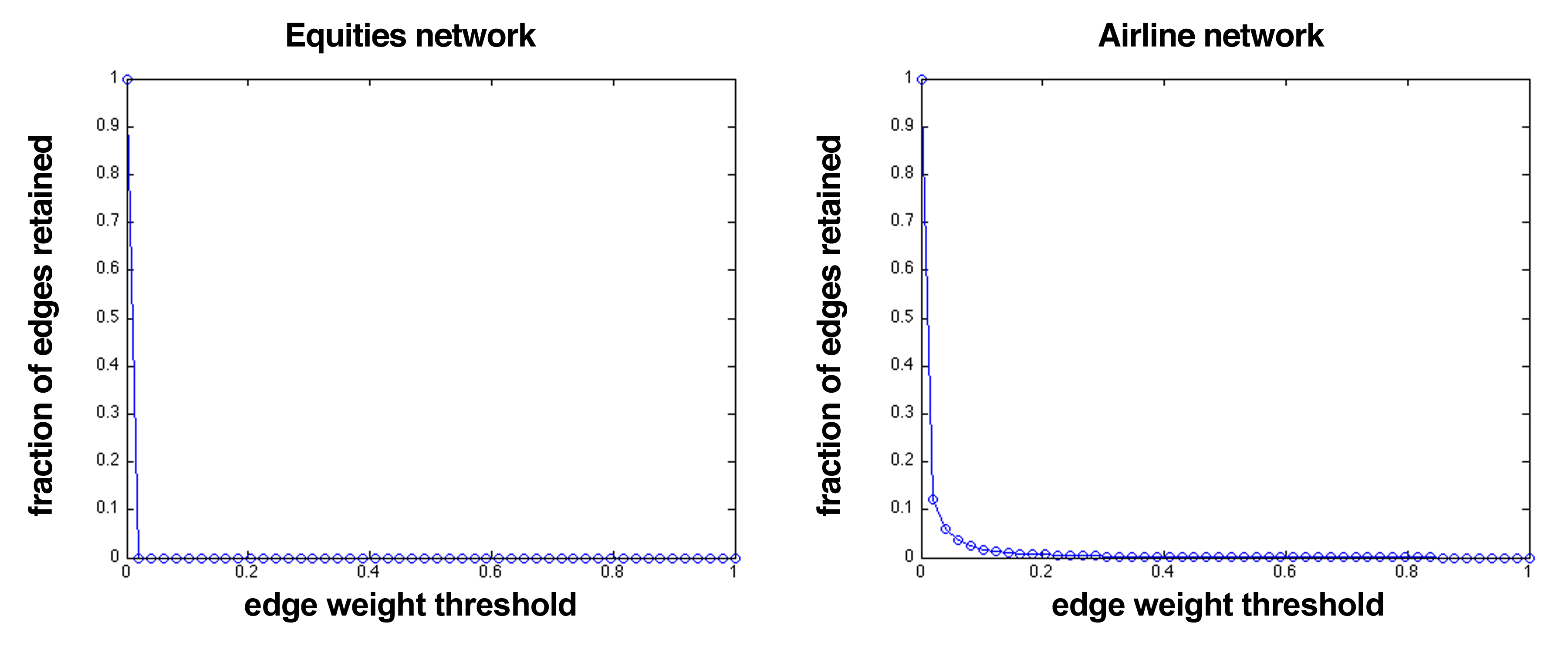

Supplement: Figure S5 — Fraction of edges retained as a function of edge weight threshold for backbone networks created using the bistochastic filter from the equities and airline networks. Note the exponential drop in the number of edges retained, indicating that the bistochastic transformation does not allow for a smooth addition of edges. (TIFF) [file pone.0016431.s006.tiff]
