# Supplementary material for: Nonparametric Sparsification of Complex Multiscale Networks
Source: PLoS One. 2011 Feb 8;6(2):e16431. doi: 10.1371/journal.pone.0016431 (PMC3035633; doi:10.1371/journal.pone.0016431)
Supplement: Table S1 — Number of Kolmogorov-Smirnov tests that rejected the null hypothesis that the empirical distributions and the parametric distributions of [5] were the same, at the and significance levels. The total number of tests for each network is given as well. We chose to compare the empirical distributions of fractional edge weight to the correct parametric cdf for all nodes in each network that had at least 40 unique nonzero edges. This number was chosen to ensure accurate results. (PDF) [file pone.0016431.s007.pdf]

	Equities	Airline	Art
# comparisons	874	173	45
fraction H_0 rejected (at 0.05)	1.0	.994	1.0
fraction H_0 rejected (at 0.01)	1.0	.983	1.0
